# Association Between Genotoxic Effects of Ageing Dental Restorations and Micronuclei in Oral Mucosal Cells

**DOI:** 10.3390/medicina61081363

**Published:** 2025-07-28

**Authors:** Csilla Benedek, Bernadette Kerekes-Máthé, Zsuzsanna Bardocz-Veres, Boglárka Szabó, Alina Iacob, Alexandra Stoica, Timea Dako, Mónika Kovács, Lóránd Dénes, Liana Bereșescu

**Affiliations:** 1 Department of Periodontology and Oral Diagnosis, Faculty of Dental Medicine, “George Emil Palade” University of Medicine, Pharmacy, Science and Technology of Târgu Mureș, 38 Gh. Marinescu Str., 540142 Târgu Mureș, Romania; csilla.bukhari@umfst.ro; 2 Department of Tooth and Dental Arch Morphology, Faculty of Dental Medicine, “George Emil Palade” University of Medicine, Pharmacy, Science and Technology of Târgu Mureș, 38 Gh. Marinescu Str., 540142 Târgu Mureș, Romania; bernadette.kerekes-mathe@umfst.ro; 3 Department of Oral Rehabilitation and Occlusology, Faculty of Dental Medicine, “George Emil Palade” University of Medicine, Pharmacy, Science and Technology of Târgu Mureș, 38 Gh. Marinescu Str., 540142 Târgu Mureș, Romania; 4 Individual Researcher, Stomatologie Mülfay Fogászat, Horea Street nr 13/A, 540051 Târgu Mureș, Romania; szabo.boglarka8@gmail.com; 5 Department of Oral and Maxillofacial Surgery, Faculty of Dental Medicine, “George Emil Palade” University of Medicine, Pharmacy, Science and Technology of Târgu Mureș, 38 Gh. Marinescu Str., 540142 Târgu Mureș, Romania; alina.iacob@umfst.ro; 6 Department of Odontology and Oral Pathology, Faculty of Dental Medicine, “George Emil Palade” University of Medicine, Pharmacy, Science and Technology of Târgu Mureș, 38 Gh. Marinescu Str., 540142 Târgu Mureș, Romania; alexandra.stoica@umfst.ro (A.S.); timea.dako@umfst.ro (T.D.); monika.kovacs@umfst.ro (M.K.); 7 Department of Anatomy and Embryology, Faculty of Medicine, “George Emil Palade” University of Medicine, Pharmacy, Science and Technology of Târgu Mureș, 38 Gh. Marinescu Str., 540142 Târgu Mureș, Romania; lorand.denes@umfst.ro; 8 Department of Preventive and Community dentistry, Faculty of Dental Medicine, “George Emil Palade” University of Medicine, Pharmacy, Science and Technology of Târgu Mureș, 38 Gh. Marinescu Str., 540142 Târgu Mureș, Romania; liana.beresescu@umfst.ro

**Keywords:** micronuclei, genotoxic effect, restoration aging, amalgam restoration, composite restoration

## Abstract

*Background and Objectives*: Dental restorations can be composed of various materials, including amalgams and methacrylate-based resins. The health risks associated with the components of the restorative materials have always been a concern, even more so with the ageing of the restorations. As the micronucleus (MN) test is a standard, accessible, and minimally invasive technique for studying the genotoxic effect of clastogenic chemicals on oral mucosal cells, the current study was conducted to determine the frequency and morphological properties of MN in the exfoliated oral mucosal cells. *Materials and Methods*: A total of 115 aged composite and amalgam restorations were included in this study. Epithelial cells were collected from the gingival tissue adjacent to the restorations of each patient and stained with a hematoxylin–eosin (HE) stain. After evaluation of the slides, the results were subjected to statistical analysis using Chi-square tests. The level of significance was set at 0.05. *Results:* The mean number of MN was significantly lower for composite restorations compared to amalgam restorations. There were no statistically significant differences between composite restorations aged 1–5 years, 5–10 years, over 10 years, and amalgam restorations aged over 10 years in the location (*p* = 0.11), staining (*p* = 0.11), or morphological characteristics (*p* = 0.18) of the MN. *Conclusions*: Despite the main limitation of this study, the lack of a control group, our results suggest that long-term exposure to restorative fillings and the ageing of these materials can cause DNA damage locally in the adjacent sites of oral cavity.

## 1. Introduction

Composite and amalgam fillings are often in direct and permanent contact with the gingival margin and therefore have a long-term impact on this soft tissue [1]. Since oral mucosal cells are in direct contact with dental materials, they are the first to be affected by any potentially harmful iatrogenic factors. The oral mucosa is subjected to high concentrations of dental restorative materials due to factors such as bacterial corrosion, variations in salivary pH, temperature changes, as well as chewing and brushing habits [2]. Every biomaterial used in dental restorations, including fillings, must undergo rigorous testing and meet strict regulatory standards before being approved for use. As a result, a high level of biocompatibility is essential for all dental materials [1,3,4]. Biocompatibility is the ability of a biomaterial to integrate into a living system without causing harmful effects or adverse reactions.

Determining the biocompatibility of a dental material requires checking several parameters: 1. cytotoxicity, 2. genotoxicity, 3. mutagenicity, 4. carcinogenicity, and 5. immunogenicity [4,5,6].

Cytotoxicity refers to the degree to which a substance can cause damage to cells, potentially leading to cell dysfunction, membrane disruption, or the induction of apoptosis. Genotoxicity refers to an agent’s ability to damage a cell’s genetic information, such as DNA or RNA. This damage can lead to genetic mutations that may induce malignant transformation [4].

Among the various factors, genotoxicity is particularly significant due to its critical implications for health and safety, especially given that DNA damage can potentially lead to cancer. This consideration is essential when evaluating materials, chemicals, pharmaceuticals, or environmental exposures, as genotoxicity assessments help determine the impact of contaminants on both public health and ecosystems [7].

The World Health Organization (WHO) has identified ten chemicals of major public health concern, one of which is mercury [8]. Elevated levels of genotoxicity have been observed in individuals exposed to mercury through dietary intake, inhalation, dermal contact in occupational settings, or from dental restorations [2]. Mercury exists in various chemical forms, each exhibiting distinct properties and toxicity levels. These are typically categorized into elemental mercury (which can damage the lungs and central nervous system when inhaled), inorganic mercury (which primarily affects the kidneys and is less readily absorbed), and organic mercury compounds, such as methylmercury and ethylmercury, which are highly toxic and mainly acquired through dietary exposure [9].

Although amalgam fillings contain mercury, this element does not easily penetrate cells. Upon exposure to the enzyme catalase—which breaks down hydrogen peroxide in the body—elemental mercury is converted into its inorganic form. This transformation reduces its lipophilic nature, thereby limiting its ability to cross cell membranes and enter cells [4].

However, if mercury does enter the body, it shows a strong affinity for binding to sulfhydryl groups, which can disrupt cellular function and damage DNA. This effect is particularly concerning in individuals with specific genetic variations that may increase their susceptibility to mercury toxicity [10,11].

The genotoxicity of mercury and its derivatives primarily stems from their ability to generate reactive oxygen species and free radicals. This oxidative stress typically occurs when mercury enters cells, either through protein transporters or directly across the plasma membrane. Concerns about the potential toxicity of mercury-containing dental amalgams have prompted numerous in vitro and in vivo studies investigating their effects on the health of individuals with amalgam fillings [4,12,13,14,15]. While both the FDI and the World Health Organization (WHO) have outlined strategies to minimize and eventually phase out the use of dental amalgam, its use in dental practice has not yet been completely banned [8].

All dental materials in the oral cavity are vulnerable to biological, physical, chemical, microbiological, thermal, and enzymatic factors; their biocompatibility might alter over time due to the release of components from the material [4].

The genotoxicity and cytotoxicity of composite dental materials are primarily attributed to incomplete polymerization reactions. This incomplete curing leads to the release of free monomers—such as TEGDMA, HEMA, UDMA, and Bis-GMA—from the organic matrix of the filling material, particularly under the influence of intraoral factors previously mentioned. These released monomers can generate reactive oxygen species (ROS), which in turn induce oxidative stress. The biological consequences include an increased frequency of micronuclei (MN), production of inflammatory mediators, disruption of the cell cycle, and the potential induction of cell necrosis and apoptosis [12,13,14].

Therefore, continuous monitoring and further research into dental materials are necessary, even after they have passed all safety tests and received regulatory approval for clinical use [4]. Several studies have reported a strong correlation between the extent of cellular damage and the age of dental fillings. In response, exfoliated buccal cells were collected near both amalgam and composite restorations for analysis [2,4].

A range of sophisticated techniques, including both in vitro and in vivo assays, are available to evaluate the toxicity of substances capable of inducing DNA damage. Among the most reliable in vivo methods are three key cytogenetic tests: the chromosomal aberration test, the comet assay, and the MN test [4,16,17,18,19]. In this study, the MN test was employed to assess the genotoxic effects of dental restorative materials on oral mucosal cells. Given that the oral mucosa is in direct and prolonged contact with these materials, its cells serve as effective biomarkers for detecting genotoxic effects [4].

The MN test is a sensitive and minimally invasive technique for detecting genetic damage. It evaluates parameters such as the frequency of MN, the number of cells containing MN, variations in the shape and size of MN, the presence of nuclear buds or bridges, and signs of chromatin condensation.

The present study aimed to evaluate the potential genotoxicity of two widely used dental restorative materials—composite and amalgam fillings—on oral mucosal cells, using the MN test.

## 2. Materials and Methods

The present study was approved by the Ethics Committee of Scientific Research of the G.E. Palade University of Medicine, Pharmacy, Science and Technology of Târgu Mureș (Approval no 2761 of 26 January 2024).

The sample size was calculated using GPower 3.1.9.7 software. A total of 115 dental restorations were performed for 87 voluntary participants, aged between 31 and 63 years, who were patients of the Odontology and Periodontology Discipline at the Faculty of Dental Medicine, George Emil Palade University of Medicine, Pharmacy, Science, and Technology of Târgu Mureș. This study was conducted between 3 March and 30 September 2024.

Inclusion criteria were: amalgam or composite dental restoration in contact with marginal gingiva, 1 year old or older than 1 year amalgam or composite fillings, completely healthy patients with no general diseases in the background, lack of prosthetic or orthodontic restoration material in the oral cavity, patients with no oral pathologies, such as: oral mucosal diseases or traumatic ulcers, and patients with no oral surgical interventions in the last 6 months.

Exclusion criteria were smoking, alcoholism, patients with general health issues, regular drug intake in the last 3 months, dental restoration having no contact with the adjacent gingival margin, recently placed amalgam or composite fillings, presence of prosthetic or orthodontic restoration material in the oral cavity, patients with oral pathologies, recent oral surgical interventions in the oral cavity conducted in the last 6 months.

Dental restorations were observed and categorized as follows:

A: composite restorations aged 1–5 years (*n* = 39; 33.92%, class II (*n* = 21; 53.85%) and class V (*n* = 18; 46.15%));

B: composite restorations aged 5–10 years (*n* = 25; 21.74%, class II (*n* = 16; 64%) and class V (*n* = 9; 36%));

C: composite restorations aged over 10 years (*n* = 34; 29.56%, class II (*n* = 29; 85.29%) and class V (*n* = 5; 14.71%));

D: amalgam restorations aged over 10 years (*n* = 17; 14.78%, class II).

As amalgam fillings have gradually become less common in Mureș county, Romania, they can now only be found in adults over the age of 40. This implies that all amalgam fillings are at least ten years old.

Epithelial cells were collected from the gingival tissue adjacent to the dental restorations. Before sampling, patients rinsed with water for 30 s to eliminate debris. The same operator (C.B.) collected all 115 samples using a sterile cytological sampling brush. The operator brushed the area of interest two to three times and then swept the brush rapidly across a clean microscopic glass slide, pressing firmly without folding, destroying, or overlapping the cells. The slides were coded and fixed. The fixing process involved adding 99% alcohol to the slide with a small, clean pipette. This preserved the sample and prevented deterioration. Complete drying required five minutes. No additional external air spray or air syringe was utilized to avoid overlapping, wrinkling, or folding the sample cells.

To reflect the oral health status of the patients, the following indices were recorded after each sampling by the same operator (C.B.): Plaque Index (PI), Silness–Löe Gingival Index (GI), and Mühlemann Papillary Bleeding Index (PBI). The measurements were performed using a UNC 15 periodontal probe. Since the sampling and the recording of the indices were carried out by the same operator, the risk of bias has been eliminated, and the reproducibility has been ascertained.

The PI records the presence of supragingival plaque on all four tooth surfaces. This index measures the thickness of plaque along the gingival margin; it is an objective clinical tool to assess oral hygiene and—subsequently—gum health. The PI can be reported as [20]:

0 = no plaque;

1 = the plaque covers 1/3 or less of the tooth surface;

2 = a maximum of 2/3 of the tooth is covered by plaque;

3 = more than 2/3 of the tooth surface is covered by plaque.

Since subjects were assured of no risk—chemicals included—no disclosing tablets have been used in the current investigation; therefore, the investigation has been conducted with the UNC 15 periodontal probe. The cervical 1/3 of the tooth has been scraped with the rounded tip of the periodontal probe, searching for plaque in this region. If no plaque remained on the tip of the probe, the Plaque Index was considered 0; otherwise, it was 1st degree. After cleaning the tip of the probe, it has been proceeded to the middle 1/3 of the tooth, scraping it. In case of a positive test, the degree was considered 2; in case of a negative test, it remained 1. The occlusal 1/3 was the last area to check with the same procedure: in case of positive findings, the degree switched to 3; otherwise remained 2.

The GI records gingival inflammation in three grades [21]:

0 = complete absence of inflammation of the gingiva;

1 = mild inflammation, discoloration;

2 = moderate inflammation, erythema, edema, bleeding on probing;

3 = severe inflammation, high degree of edema, cyanotic gums, spontaneous bleeding.

Gingival Index has been recorded through inspection.

Levels of PBI were evaluated 20–30 s after periodontal probing, according to Saxer and Mühlemann [20]. The PBI discriminates five different degrees (intensities) of bleeding after carefully probing the gingival sulcus in the papillary region. The PBI can be reported as:

0 = no bleeding on gentle probing;

1 = punctate bleeding occurs when probed;

2 = line-like bleeding or appearance of several isolated bleeding points on probing;

3 = the interdental triangle is filled with blood;

4 = heavy bleeding occurs, filling the interdental space and spreading along the sulcus in the form of a drop.

To record the PBI, the rounded tip of the periodontal probe has been placed on the tip of the distal interdental papilla of the tooth in discussion, a sliding movement has been conferred to it, maintaining contact with the soft tissue, until the mesial interdental papilla’s tip. No pressure has been exerted, only gentile contact has been maintained, to not alter the results.

In the next stage, the samples were processed for cytology. The glass plates were placed in an upright glass slide holder and washed with 70% alcohol and distilled water for 10 min. The samples were stained with hematoxylin–eosin (HE) dye. First, the nuclei were stained with hematoxylin for 5–10 min to give a blue to purple color. Several rinses with distilled water for 1–2 min followed this to remove excess dye. Second, eosin was used for 30 s–2 min to stain the cytoplasm pink, followed by rinsing with distilled water for an additional 1–2 min and drying for 5–10 min.

The number of MN and other morphological alterations in the nucleus was counted in the cells using the MN test. The analysis was performed at 400× magnification using an optical microscope (Zeiss, Oberkichen, Germany), connected to an operational camera to capture results. Each MN and other chromatin abnormalities were also examined at 1000× magnification. Two hundred epithelial cells were examined for every participant, and Tolbert’s criteria [22,23] were followed for their evaluation. Criteria used for cell selection and cell identification:

1. Intact cytoplasm and relatively flat cell position on the slide;

2. Slight or no overlap with neighboring cells;

3. Little or no debris;

4. Normal, intact, smooth, and distinct nuclei.

A cell considered to be a MN must meet the following criteria [24,25]:

1. Round shape, which refers to a membrane;

2. Less than one-third the size of the nucleus, but large enough to be noticeable;

3. Color and staining: the texture and staining intensity are similar to those of the cell nucleus;

4. Position: it is on the same focal plane as the nucleus;

5. Not shiny or refractile;

6. No overlap or bridging with the nucleus.

**Figure 1:**
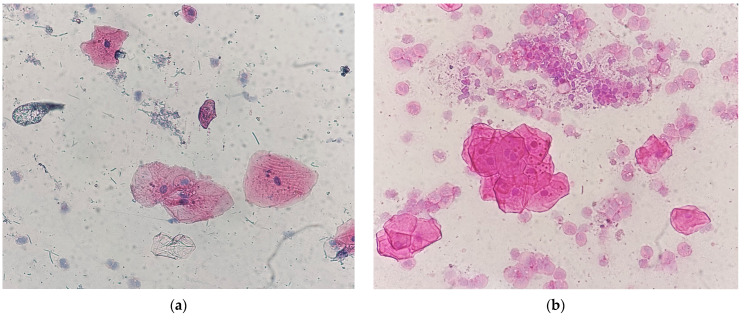
Gingival epithelial cells, HE staining, 40× (**a**) less than 5 MN; (**b**) 5–10 MN.

The samples were evaluated based on the following morphological changes: occurrence, number, shape, location, and staining of MN. The occurrence of MN was expressed as a percentage of the 200 cells that had MN on the examined plate. Based on the number of MN, samples were divided into two categories: less than 5 MN (Figure 1a) or between 5 and 10 MN within a cell (Figure 1b).

**Figure 2:**
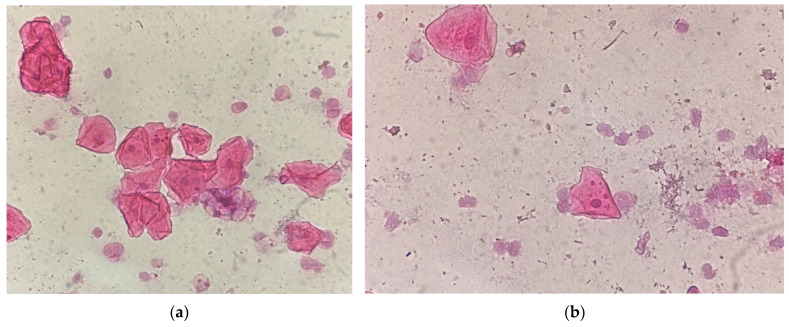
Gingival epithelial cells, HE staining, 40× (**a**) MN of the same size; (**b**) MN of different sizes.

**Figure 3:**
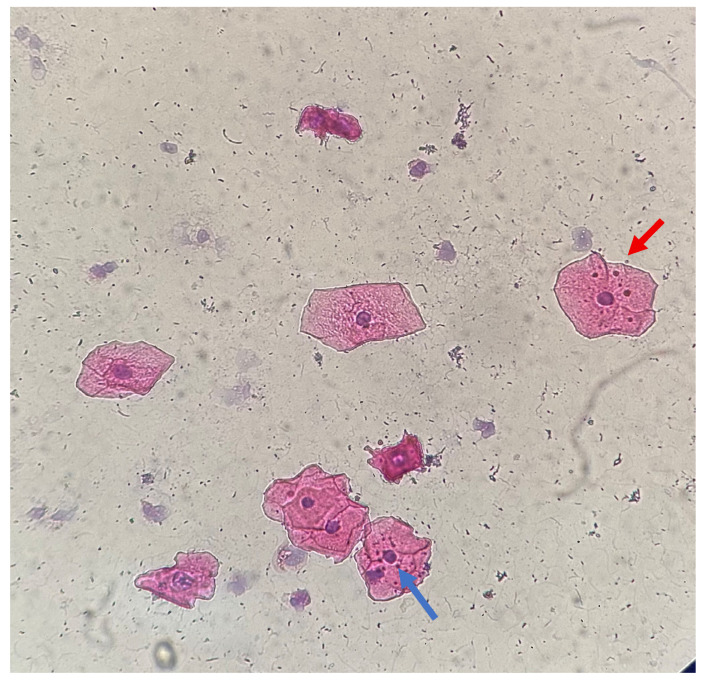
Gingival epithelial cells, HE staining, 40×: MN with different locations—red arrow shows MN located in the cytoplasm, far from the nucleus, while the blue arrow shows MN in contact with the nucleus.

**Figure 4:**
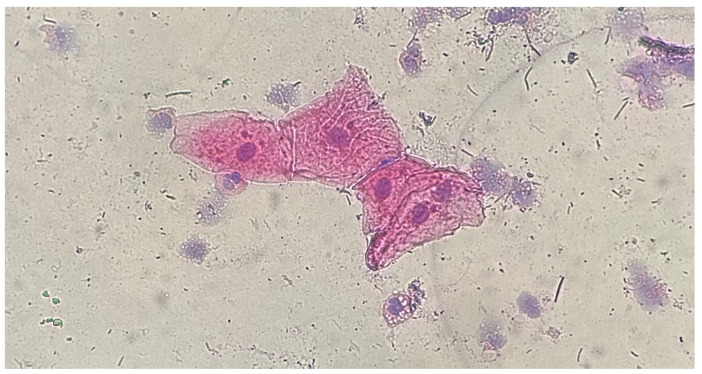
Gingival epithelial cells, HE staining, 40×: MN of the same staining as the cell nucleus.

**Figure 5:**
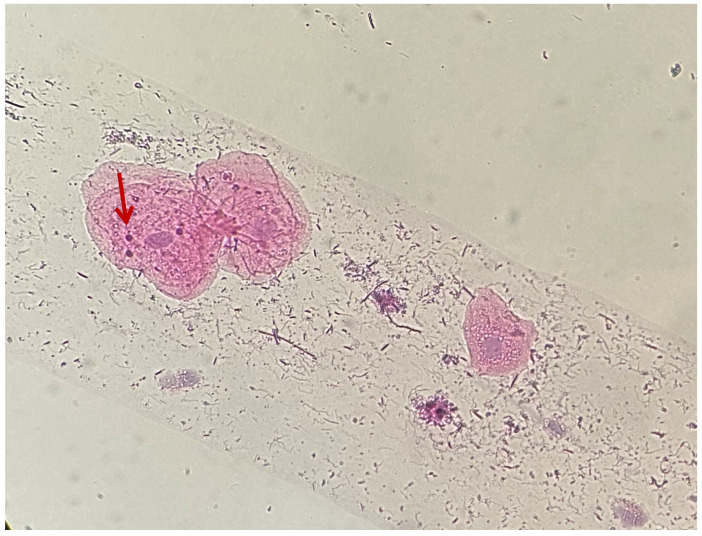
Gingival epithelial cells, HE staining, 40×: hyperchromatic MN (red arrow).

Differences in size (Figure 2a,b), location (Figure 3), and shape of the MN within a cell were observed. Staining of MN was compared with the staining of the cell nucleus (Figure 4 and Figure 5).

The data obtained were summarized in an Excel table. GraphPad Prism 9 was used for statistical analysis, performing Chi-square tests. The level of significance was set at 0.05.

## 3. Results

The recorded indices and their percentage for each restoration group are presented in Table 1.

Regarding the occurrence of micronuclei, they were present in a smaller percentage next to composite fillings older than 10 years (36%) compared to those between 1 and 5 years (45%) and 5 and 10 years (43%). There is a slight decreasing trend with the progression of years. In the case of amalgam fillings, 49% of the cells contained micronuclei. When examining the number of micronuclei, a higher percentage of samples showed less than 5 micronuclei for composite fillings. Among the amalgam restoration samples, 86% had 5–10 micronuclei within a single cell (Table 2).

Examining the morphological properties of micronuclei, a higher percentage of amalgam fillings showed micronuclei of different sizes within a single cell compared to composite fillings, where micronuclei were predominantly of uniform size within a single cell. Regarding the location of micronuclei, for both amalgam and composite fillings, a higher percentage of micronuclei clustered around the nucleus, with fewer scattered in the cytoplasm. Regarding the staining of micronuclei, for both amalgam and composite fillings, a higher percentage of micronuclei had the same staining as the nucleus, while fewer were hyperchromatic (Table 3).

There were no statistically significant differences between groups in terms of the location (*p* = 0.11), staining (*p* = 0.11), or morphological properties (*p* = 0.18) of micronuclei. There was a statistically significant difference in the occurrence of micronuclei in percentage between the amalgam restoration group with 5–10 micronuclei (86%) and the composite restoration group with 5–10 micronuclei (24% on average) (*p* = 0.00001).

## 4. Discussion

The current study used a MN assay to investigate the occurrence and morphological alterations of MN in composite restorations aged 1–5 years, 5–10 years, and above 10 years, as well as amalgam fillings aged 10 years or more. Swaps were only taken adjacent to the fillings.

There are several precise screening methods to assess genotoxicity, including cytogenic tests (chromosomal aberration assay, MN assay), prokaryotic cell-based strategies (vitotox assay, etc.), and modern molecular strategies (tests for biomarker identification, etc.). The MN assay is currently recognized as one of the most effective and reliable methods for detecting genotoxic carcinogens. This test is based on the appearance of MN in treated cells [26,27].

In the oral cavity, a simpler but effective method relies on the formation of MN in exfoliated epithelial cells, known as MN analysis, which is performed on oral epithelial cells to measure their frequency. This is a widely used biomarker for assessing cytogenetic damage. Their investigation began in the 1980s, as the oral cavity was considered the most sensitive to genotoxic agents [28].

The advantages of MN analysis are its relative ease of counting, low cost, noninvasive sampling, and relevant results due to the evaluation of a large number of cells [29].

Nersesyan et al. studied the incidence of MN simultaneously in buccal cells and lymphocytes, utilizing two different methods: the accurate cytokinesis-block method in lymphocytes and the less invasive MN analysis in buccal cells. The findings of the study show that data acquired using the buccal MN assay are consistent with those obtained using the lymphocyte cytokinesis-block MN assay. This means that the buccal MN assay also identifies people with a higher risk of developing tumors [30].

The present study aimed to analyze MN near composite and amalgam dental fillings, which were in direct contact with the marginal gingiva. To achieve this, the MN test was conducted to assess the frequency of MN in oral exfoliated epithelial cells collected from the specified area.

Previous studies have shown that duration of exposure plays a significant role in the cytotoxic and genotoxic changes associated with dental materials [2,31,32]. Therefore, only cases with amalgam and composite fillings that had been in place for over one year were included in this study.

The presence of MN has been widely used as a biomarker for genotoxic stress and genomic instability across a broad range of human and non-human models [33,34]. MN frequently exhibit defects in their nuclear envelope—an essential barrier that protects the genome from the cytoplasmic environment and regulates nucleocytoplasmic transport. Disruption of the nuclear envelope can result in DNA damage within MN due to impaired DNA replication and repair, as well as the exposure of interphase chromatin to the cytoplasm [35,36,37].

MN arise from lagging chromosomes or acentric chromosome fragments [38,39,40,41,42,43] that fail to integrate into daughter nuclei and are instead encapsulated by a separate—often atypical—nuclear envelope [44,45,46]. While defects in spindle assembly have long been recognized as contributors to MN formation, recent studies have identified additional molecular factors involved. These include dysfunctional centromeres and kinetochores, aberrant kinetochore–microtubule interactions, and defects in mitotic spindle formation, all of which contribute to chromosome misaggregation and slippage [47]. Different lines of evidence have pointed to MN as a source of recently portrayed complex genome rearrangements, including chromothripsis, which could be a trademark of numerous cancer types [37].

Chromosomal instability, a trademark of forceful cancers, is characterized by the presence of MN, which are cytosolic rupture-prone structures that contain whole chromosomes or chromosome arms. The irreversible collapse of micronuclear envelopes may be a key event in tumor evolution. Micronuclear collapse displays the DNA to the cytosol, catalyzing chromosomal rearrangements and epigenetic changes that affect tumor heterogeneity as well as treatment resistance. Micronuclear burst triggers inflammatory signaling pathways as well, which reshape the tumor-resistant microenvironment, advancing metastasis [48].

Given these implications, MN analysis plays a critical role in patient screening and may serve as an early indicator of malignant transformation. Therefore, individuals receiving synthetic materials in the oral cavity (e.g., dental fillings, bleaching agents, and prosthetic devices) or those with harmful habits such as smoking should undergo regular MN testing for early detection and monitoring.

In the present study, composite fillings older than 10 years showed a lower frequency of MN (36%) compared to those aged 1–5 years (45%) and 5–10 years (43%), indicating a slight decreasing trend over time. In contrast, amalgam fillings exhibited a higher MN frequency (49%) than all composite categories.

From the point of view of the number of MN within one cell, in our study, the composite fillings showed mainly cells with less than 5 MN; on the contrary, in amalgam fillings, 86% of microscope slides showed a dominant number of MN between 5 and 10 within a cell. Both findings show that mercury from amalgam fillings is more toxic than the composite fillings. Reichl et al. [4,49] came to the same conclusion, based on their in vitro study regarding the behavior of the dental amalgam and composite on the human gingival fibroblasts. Based on the current literature, mercury can enter the living body only with difficulty, but time can change this characteristic, having a strong impact on the biocompatibility of the dental materials. Mary et al. detailed that in their study, the number of MN within the cells of amalgam filling was measurably significantly higher than that of composite fillings [2,31].

Similar to our study, Gavic and his team examined MN adjacent to Class II restorations in 60 children. They did not take a control sample from the adjacent mucosa of the non-filled region, but followed the development of MN over time after the filling had been placed. They found that the MN number stabilized 90 days after filling, the results have not shown any further significant differences in the time lapse [50].

Our paper suggests that there is a slight decreasing trend with the progression of years, after the MN number stabilized.

Although the present study lacks the swaps around healthy teeth for comparison, it is nevertheless crucial to indicate the average MN number based on the specialty literature.

The mean MN number in the buccal mucosa or gingival epithelium adjacent to healthy, non-filled teeth in healthy individuals serves as a comparison in genotoxicity studies. There are solid, peer-reviewed figures for mean MN frequency in epithelial cells adjacent to healthy, non-filled teeth.

Mary et al. sampled MN from amalgam and composite restorations, but since they were examining recently placed restorations, the first sample was obtained prior to the filling. The mean number of MN per 500 cells before the amalgam restoration was reported as being 1.05 ± 0.75, which increased to 5.41 ± 1.25, and similarly, before and after the composite restoration mean number of MNs was 0.96 ± 0.50 and increased to 2.83 ± 0.85 [2].

In a cross-sectional study by Tadin et al., involving 35 patients with chronic periodontitis and 30 healthy individuals as a control group, the average number of MN observed was 1.8 ± 1.49 per 1000 cells in the periodontitis group and 2.0 ± 1.34 per 1000 cells in the healthy control group. The difference between the groups was not statistically significant [51].

A baseline from a meta-analysis of MN frequency in buccal mucosal cells indicated a mean MN frequency of 1.10 per 1000 cells [52].

According to the place of occurrence in the oral cavity, it can be stated that, in case of gingival epithelium, the average MN frequency per 1000 cells is considered to be 2.00 ± 1.34, and in buccal mucosal cells, 1.10 (95% CI: 0.70–1.72) [51,52].

As a summary of baseline levels used in genotoxicity comparisons, for healthy individuals with no restorations, non-smokers, and good oral hygiene, the MN frequency in exfoliated buccal/gingival epithelial cells is generally between 0.5 and 2.5 MN per 1000 cells [53,54]. Slightly broader ranges might occur, depending on the staining method, population, and microscope calibration [51,52,53,54].

In our study, the shape analysis of MN revealed that amalgam fillings exhibited a higher percentage of cells containing MN of varying sizes (57%), in contrast to composite fillings, where MN within a single cell were predominantly uniform in size. This finding highlights the genotoxic potential associated with mercury in amalgam restorations.

Ahmed et al. reported that the cytotoxic and genotoxic effects of composite fillings increase over time as the material remains in the oral cavity. In contrast, amalgam fillings demonstrate a peak in cytotoxic and genotoxic activity within a few hours after placement, followed by a gradual decline [55].

More recently, researchers such as Caministeanu et al., Dhar et al., Alsahli et al., and others have emphasized that the selection of dental materials should be guided by evidence-based scientific research to ensure patient safety and minimize adverse biological effects [56,57,58].

Regarding the location of MN, in both amalgam and composite fillings, a higher percentage of MN were clustered around the nucleus, and a lower percentage were scattered in the cytoplasm.

Examining the staining of MN in both amalgam and composite fillings, a higher percentage of MN with the same staining as the cell nucleus occurred, and a lower percentage of hyperchromic MN were present. The features mentioned above indicate that both amalgam and composite fillings induce genome damage, inducing morphological changes in the cells.

In the present study, the Papillary Bleeding Index (PBI)—a key indicator of inflammation—revealed that approximately 30% of sites around composite fillings exhibited bleeding, while 42.9% of sites around amalgam fillings showed a positive bleeding response. This outcome is closely associated with both the technical accuracy of the restoration placement by the clinician and the patient’s oral hygiene practices.

The primary limitation of this study is the absence of a control group consisting of samples collected from areas adjacent to intact, healthy teeth.

This study was designed without a control group because the primary interest was not in how many MN formed in a particular location where a filling was subsequently placed, but rather in comparing the occurrence of MN in fillings of a certain type and age. A control group, in our opinion, would have been required primarily if follow-up fillings had been performed: to place a filling in a previously filling-free site and to monitor the development of MN prior to and after the filling was placed for a well-defined length of time.

The current study was not intended to track follow-up fillings, but rather to monitor fillings placed at different times. As a result, the emphasis is on how the quantity of MN evolves with the age of the filling, rather than how they form from nearly nothing. However, if a control group had existed within the same oral cavity, using swaps from an untreated zone, the results would have been more noticeable. However, this gap allows for future inquiry.

While this study provides valuable insights, the absence of a proper control group reduces the strength of causal inferences. Anyhow, future research would benefit from including a control group to better isolate the effects of the intervention.

Additional limitations include the lack of consideration for potential confounders, the absence of longitudinal or causative analysis, and the omission of cytological calibration parameters.

To enhance the reliability and validity of future research, we propose several improvements: increasing the sample size; including intraoral controls from non-restored regions of the same oral cavity; and adopting more advanced staining methods such as Giemsa, acridine orange, or fluorescent dyes. DNA-specific dyes such as acridine orange should be preferred over other dyes, thus reducing the false detection of MN [59,60]. The use of higher magnification microscopes and the examination of a larger number of cells per slide are also recommended. According to Tolbert et al., analyzing at least 1000 cells provides more accurate and statistically robust results [61,62].

Future studies could involve a larger number and type of restorations, including a control sample from the same oral cavity, a longer observation period, utilization of more indices and questionnaires to outline potential confounders, utilization of different staining procedures, multiple operators, or multicenter samples, assessing different types of biomaterials (GIC, etc.), utilization of different assays for MN (comet assay, etc.), extend this study for a larger region or for pediatric dentistry as well.

In conclusion, while this study provides important insights into the occurrence and morphological variations in MN in the case of long-term and different types of fillings, it should be interpreted with caution due to the lack of control swaps of a non-treated area of the same oral cavity.

## 5. Conclusions

The present study, which aimed to observe and compare micronucleus assay among participants with different ageing stages of amalgam and composite restorations, revealed that buccal cells of the subjects with amalgam fillings showed the highest degree of genotoxic changes, followed by those of composite fillings. Our results suggest that restorative dental fillings produce DNA damage locally in the oral cavity probably due to the release of mercury or methacrylate or other components thus increasing the risk of pre-cancerous and cancerous oral pathology. Within this study’s limitations, the materials’ effects are indicative. Future research should include a larger study group, and control sampling from healthy teeth in same subjects.

## Figures and Tables

**Table 1:** The recorded indices and their percentage for each group of restoration.

Type of Restoration	Type of Index	Index Level	Occurrence in Percentage (Absolute Value)
1–5 years Composite	PI	0	17.95% (*n* = 7)
		1	41.03% (*n* = 16)
		2	41.03% (*n* = 16)
	GI	0	25.64% (*n* = 10)
		1	48.72% (*n* = 19)
		2	25.64% (*n* = 10)
	PBI	0	58.97% (*n* = 23)
		1	23.08% (*n* = 9)
		2	5.13% (*n* = 2)
		3	12.82% (*n* = 5)
5–10 years Composite	PI	0	44.00% (*n* = 11)
		1	28.00% (*n* = 7)
		2	28.00% (*n* = 7)
	GI	0	40.00% (*n* = 10)
		1	36.00% (*n* = 9)
		2	24.00% (*n* = 6)
	PBI	0	60.00% (*n* = 15)
		1	12.00% (*n* = 3)
		2	20.00% (*n* = 5)
		3	8.00% (*n* = 2)
>10 years composite	PI	0	17.65% (*n* = 6)
		1	64.71% (*n* = 22)
		2	17.65% (*n* = 6)
	GI	0	5.88% (*n* = 2)
		1	82.35% (*n* = 28)
		2	11.76% (*n* = 4)
	PBI	0	70.59% (*n* = 24)
		1	11.76% (*n* = 4)
		2	17.65% (*n* = 6)
Amalgam	PI	0	17.65% (*n* = 3)
		1	29.41% (*n* = 5)
		2	52.94% (*n* = 9)
	GI	0	11.76% (*n* = 2)
		1	52.94% (*n* = 9)
		2	35.29% (*n* = 6)
	PBI	0	52.94% (*n* = 9)
		1	5.88% (*n* = 1)
		2	41.18% (*n* = 7)

**Table 2:** Number of micronuclei.

Type of Restoration	Number of Micronuclei	Occurrence in Percentage
1–5 years composite	<5	69%
	5–10	31%
5–10 years composite	<5	82%
	5–10	18%
>10 years composite	<5	77%
	5–10	23%
Amalgam >10 years	<5	14%
	5–10	86%

**Table 3:** Properties of micronuclei.

Type of Restoration	Size of Micronuclei	Occurrence in Percentage	Location of Micronuclei	Occurrence in Percentage	Staining of Micronuclei	Occurrence in Percentage
1–5 years Composite	Uniform sizes	87%	Around the nucleus	94%	Same as the nucleus	81%
	Different sizes	13%	Far from the nucleus	6%	Hyperchromatic	19%
5–10 years Composite	Uniform sizes	64%	Around the nucleus	91%	Same as the nucleus	82%
	Different sizes	36%	Far from the nucleus	9%	Hyperchromatic	18%
>10 years Composite	Uniform sizes	69%	Around the nucleus	77%	Same as the nucleus	85%
	Different sizes	31%	Far from the nucleus	23%	Hyperchromatic	15%
Amalgam	Uniform sizes	43%	Around the nucleus	57%	Same as the nucleus	100%
	Different sizes	57%	Far from the nucleus	43%	Hyperchromatic	0

## Data Availability

The dataset analyzed during this study are available from the first author on request.

## Abbreviations

The following abbreviations are used in this manuscript:

MN PI	Micronuclei Plaque Index
GI	Silness–Löe Gingival Index
PBI	Mühlemann Papillary Bleeding Index
HE	hematoxylin–eosin
